# Repeated COVID-19 vaccine boosters elicit variant-specific memory B cells in humans

**DOI:** 10.1016/j.celrep.2026.117052

**Published:** 2026-03-07

**Authors:** M. Alejandra Tortorici, Kaitlin R. Sprouse, Amin Addetia, Jack T. Brown, Jimin Lee, Cameron Stewart, Benjamin Merz, Alex Harteloo, Anna Elias-Warren, Helen Y. Chu, David Veesler

**Affiliations:** 1 Department of Biochemistry, University of Washington, Seattle, WA 98195, USA; 2 Howard Hughes Medical Institute, University of Washington, Seattle, WA 98195, USA; 3 Division of Allergy and Infectious Diseases, University of Washington, Seattle, WA 98195, USA

**Keywords:** COVID-19 vaccines, SARS-CoV-2 spike glycoprotein, immune imprinting, antigenic sin, memory B cells, serum antibodies, neutralizing antibodies, coronaviruses, Immunology, virology

## Abstract

The first exposure to a pathogen impacts subsequent immune responses towards related pathogens. This immune imprinting explains that infection or vaccination with circulating SARS-CoV-2 variants primarily recalls cross-reactive memory B cells induced by prior Wuhan-Hu-1 spike (S) exposure. The magnitude and persistence of immune imprinting in mRNA vaccinees are not understood. We investigate serum antibody and memory B cell responses after administration of multiple XBB.1.5 and JN.1/KP.2 COVID-19 vaccine boosters. We find that the JN.1/KP.2 booster elicit broadly neutralizing antibody responses against recent SARS-CoV-2 variants by recalling Wu S-induced immunity in all but one individual. We detect an increased fraction of serum antibodies, and particularly memory B cells, recognizing XBB.1.5 and KP.2, but not Wu, relative to individuals who received a single XBB.1.5 booster. Repeated exposures to antigenically divergent S thus contribute to overcoming immune imprinting and support vaccine updates for continued protection.

## eTOC

SARS-CoV-2 Wuhan-Hu-1 (Wu) S- induced immune imprinting is prevalent. Tortorici et al. show that administration of multiple XBB.1.5 and JN.1/KP.2 vaccine boosters primarily recalls Wu S-reactive serum antibodies and memory B cells. However, detection of humoral responses targeting updated vaccine antigens but not Wu suggests progressive overcoming of immune imprinting.

## Graphical Abstract

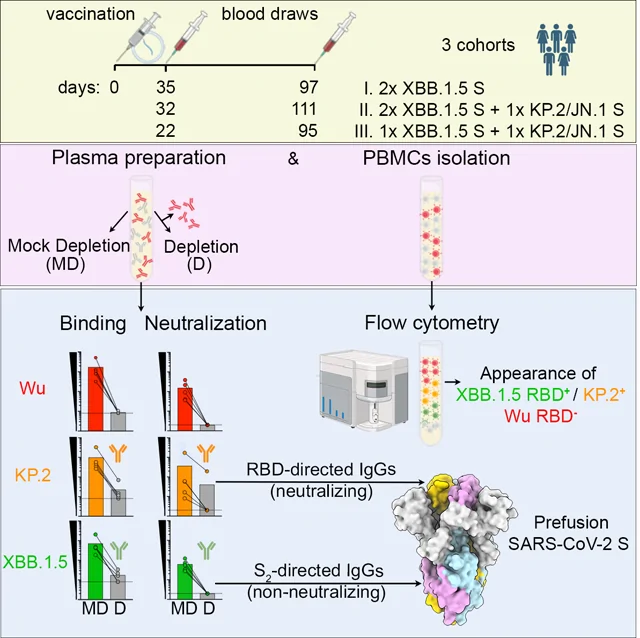

## Introduction

Severe acute respiratory syndrome coronavirus 2 (SARS-CoV-2) evolves under the selective pressure of host immunity resulting from human infection and vaccination. As a result, immune evasive variants continue to emerge with spike (S) mutations eroding polyclonal plasma neutralizing antibody titers and leading to waves of (re)infections^1–8^. This requires periodic reformulation of COVID-19 vaccines to match the S sequences of circulating SARS-CoV-2 variants and maximize their effectiveness, such as the Novavax Nuvaxovid JN.1 S (protein subunit) and the Pfizer Comirnaty and Moderna Spikevax KP.2 S (mRNA) boosters. S-directed neutralizing antibodies are a primary correlate of protection against COVID-19^9–12^, with the receptor-binding domain (RBD) as the main target of serum neutralizing activity^13–16^. It is therefore essential to assess the potency and breadth of serum antibodies upon vaccine update.

Since the emergence of the first Omicron variants late 2021^17^, it became apparent that humoral immune responses to these highly divergent SARS-CoV-2 lineages are dominated by recall of pre-existing immunity originating from repeated exposure to the ancestral Wu S glycoprotein. This phenomenon is called immune imprinting or original antigenic sin and was first noticed in humans infected with influenza virus^18^. The impact of immune imprinting on vaccine-elicited antibody responses in humans after release of the JN.1 and KP.2 COVID-19 vaccine boosters remains unknown, limiting our understanding of the contributions of updated antigens to humoral immunity.

To understand the persistence of immune imprinting, we studied serum antibody and memory B cell responses in humans who received multiple Wu S vaccine doses prior to administration of the updated XBB.1.5, JN.1 and KP.2 COVID-19 vaccine boosters. We found that the JN.1 and KP.2 vaccine boosters elicited broadly neutralizing serum antibodies with activity against Wu S and currently circulating variants coming from a recall of pre-existing humoral immunity induced by prior Wu S exposure. We detected serum antibody and particularly memory B cells recognizing the XBB.1.5 and KP.2 S antigens, but not Wu, suggesting that repeated exposures to antigenically divergent S glycoproteins through vaccine updates elicit *de novo* humoral responses and progressively overcome immune imprinting.

## Results

### Updated vaccine boosters elicit broadly neutralizing antibodies against circulating SARS-CoV-2 variants

**Figure 1.**
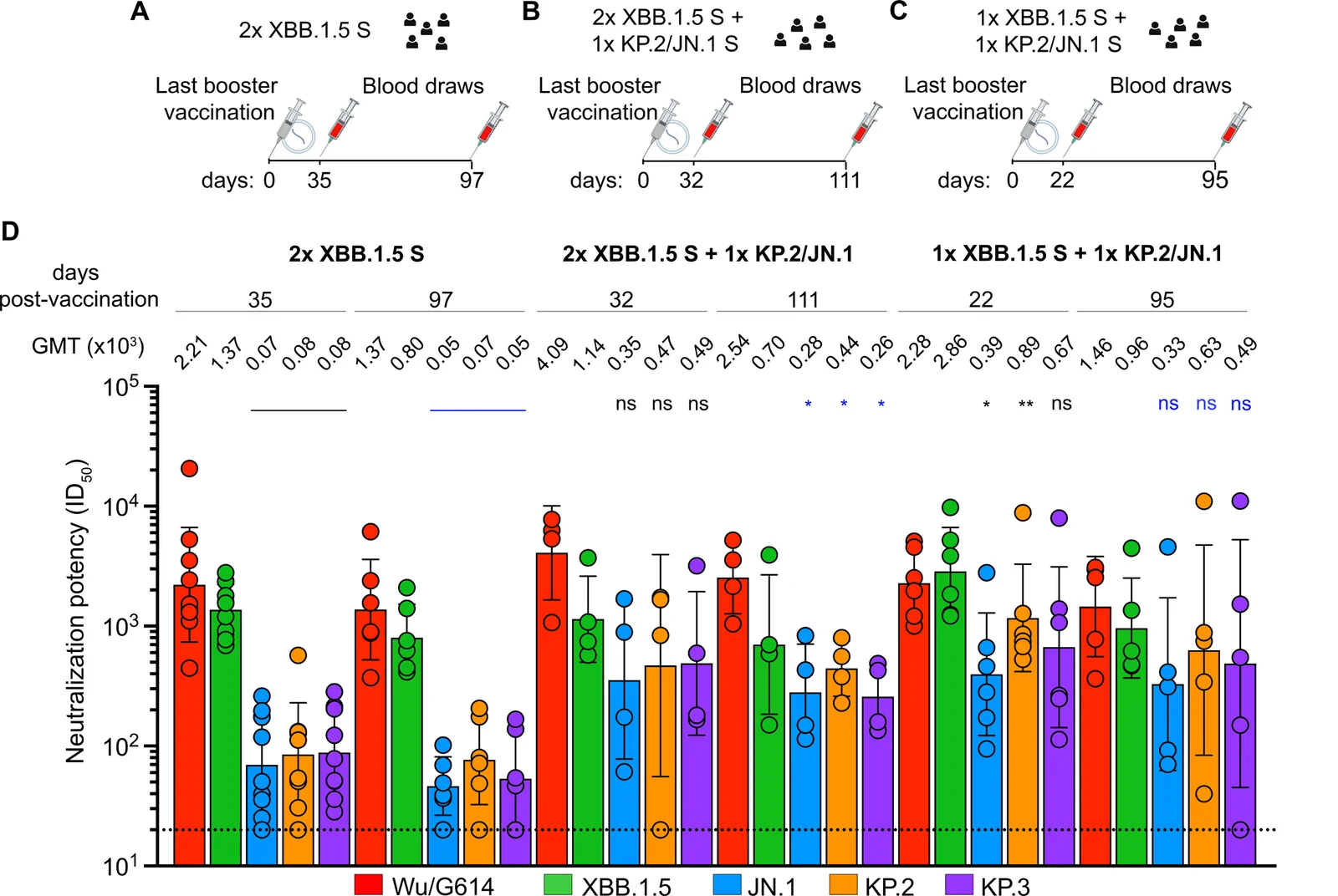
Serum neutralizing antibody responses elicited by XBB.1.5, JN.1 or KP.2 COVID-19 booster vaccination in humans. **A-C.** Timeline of vaccination and blood draws for the 2x XBB.1.5 S (A) 2x XBB.1.5 S +1x KP.2/JN.1 S (B) and 1x XBB.1.5 S + 1x KP.2/JN.1 S (C) cohorts analyzed in this manuscript. **D.** Neutralizing antibody titers evaluated using VSV pseudotyped with Wu/G614 (red), XBB.1.5 (green), JN.1 (blue), KP.2 (orange) or KP.3 (purple) S using sera obtained 32–60 days (median: 35) and 87–163 days (median: 97) from 2x XBB.1.5 S cohort (A), 31–33 days (median: 32) and 95–134 days (median: 111) from 2x XBB.1.5 S + 1x KP.2/JN.1 S cohort (B) or at 12–33 days (median: 22) and 79–123 days (median: 95) from 1x XBB.1.5 S + 1x KP.2/JN.1 S cohort (C). Each data point represents the half-maximal inhibitory dilution (ID_50_) for an individual obtained from averaging two biological replicates. Geometric mean titers (GMTs) for each cohort against each pseudotyped virus are indicated above the corresponding bar graph with error bars representing the geometric SD. The horizontal dotted dashed line indicates the limit of detection (1/20 serum dilution). Statistical analysis was performed by comparing individual ID_50_ values for each pseudovirus JN.1, KP.2 or KP.3) to the 2x XBB.1.5 S cohort from the first (black labels) and second (blue labels) blood draws independently with one-way analysis of variance (ANOVA). Not significant (ns)=P≥0.05, *P=0.01 to 0.05 and **P=0.001 to 0.01. See also Figure S1.

To evaluate the impact of the updated COVID-19 vaccine boosters on serum neutralizing activity, we analyzed serum samples from individuals with prior infection and COVID-19 vaccination. Samples from individuals were collected at 32–60 days (median: 35) and 87–163 days (median: 97) after vaccination with two doses of XBB.1.5 S (2x XBB.1.5 S cohort, Figure 1A), at 31–33 days (median: 32) and 95–134 days (median: 111) after receiving a KP.2 or a JN.1 S booster from individuals who previously received two doses of XBB.1.5 S booster (2x XBB.1.5 S + 1x KP.2/JN.1 S cohort, Figure 1B) and at 12–33 days (median: 22) and 79–123 days (median: 95) after receiving a KP.2 or a JN.1 S booster from individuals who previously received one dose of XBB.1.5 S booster (1x XBB.1.5 S + 1x KP.2/JN.1 S cohort, Figure 1C and Table 1). The neutralization potency and breadth of polyclonal serum antibodies was assessed with a vesicular stomatitis virus (VSV) pseudotyped with the Wuhan-Hu-1/G614 (Wu/G614), XBB.1.5, JN.1, KP.2 or KP.3 S glycoproteins (Figures 1, S1 and Table 2). Approximately one month following KP.2/JN.1 S booster administration, serum neutralizing activity was comparable or greater for all pseudoviruses evaluated relative to subjects that only received the XBB.1.5 S booster. Geometric mean titers (GMTs) against Wu/G614, XBB.1.5, JN.1, KP.2 or KP.3 S VSV were 2,210; 1,370, 70, 80 and 80 for vaccinees in the 2x XBB.1.5 S cohort, 4,090; 1,140; 350; 470 and 490 for the 2x XBB.1.5 S + 1x KP.2/JN.1 S cohort, and 2,280; 2,860; 390, 890 and 670 for the 1x XBB.1.5 + 1x KP.2/JN.1 S cohort (Figure 1D). Approximately three months after booster vaccination, these trends in serum neutralizing activity remained largely similar with a moderate waning of neutralizing activity (Figure 1D), as previously described^19–21^.

Our data suggest that administration of the updated KP.2/JN.1 vaccine booster elicits superior serum neutralizing activity than XBB.1.5 boosters against currently circulating variants, including mismatched pseudoviruses, as supported by absolute neutralizing activity and the more balanced ratios of neutralizing antibody titers of Wu/G614 and variants. These results support vaccine updates to match circulating strains.

### Updated booster vaccination primarily recalls Wu S-directed neutralizing antibodies

**Figure 2.**
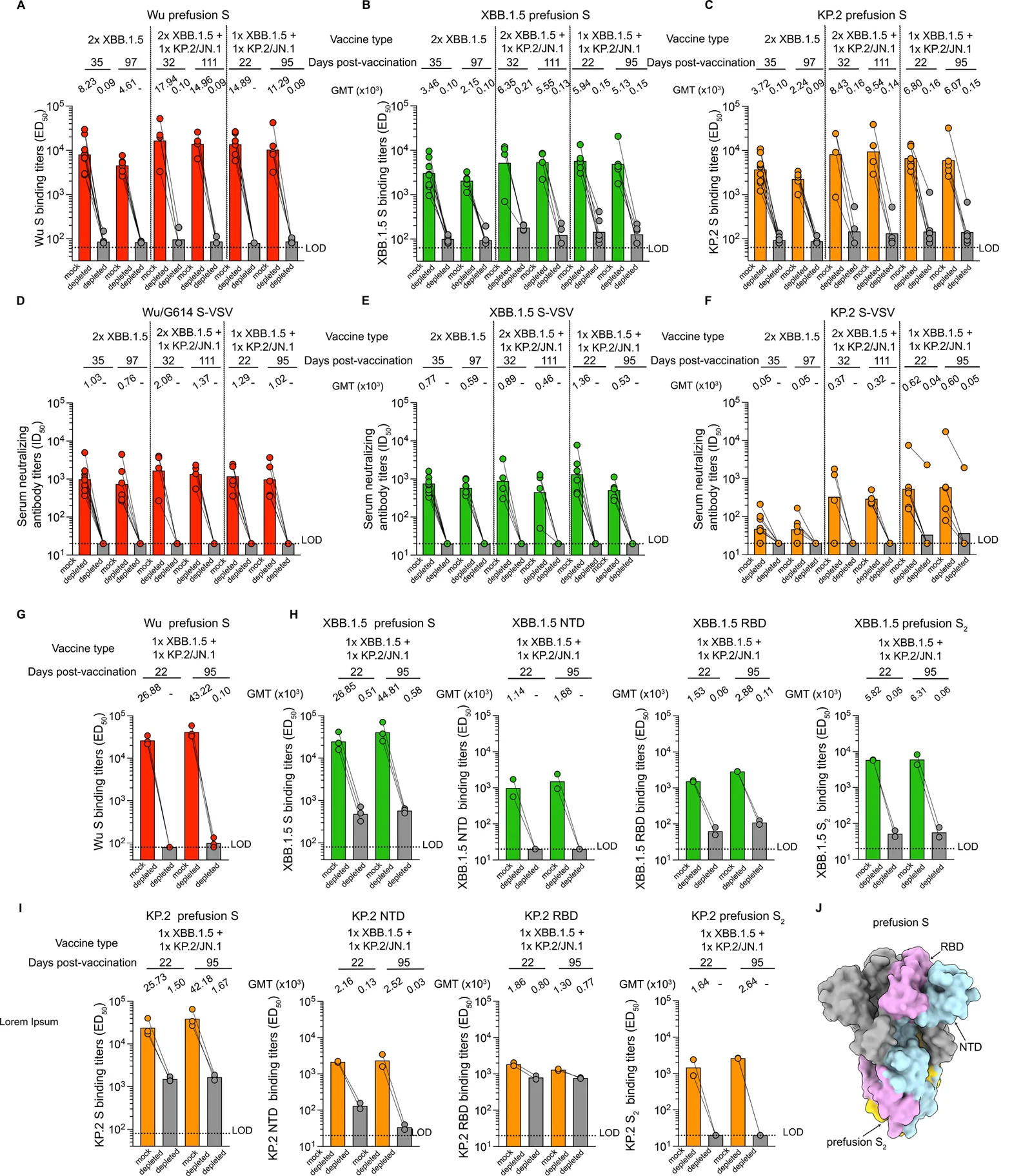
XBB.1.5 and KP.2 serum neutralizing activity is abrogated upon depletion of Wu S-reactive antibodies in all but one individual. (A-C) Antibody binding titers, expressed as half-maximal effective dilution (ED_50_), against prefusion-stabilized Wu (A) XBB.1.5 (B) and KP.2 (C) HexaPro S in the serum of individuals from the 2x XBB.1.5 S cohort (n= 15 samples), 2x XBB.1.5 S + 1x KP.2/JN.1 S cohort (n= 8 samples) or 1x XBB.1.5 S + 1x KP.2/JN.1 cohort (n= 11 samples) determined by ELISA upon mock depletion or depletion of Wu S-reactive antibodies. Limit of detection, LOD: 80. (D-F) Neutralizing antibody titers, expressed as half-maximal inhibitory dilution (ID_50_), against Wu/G614 S VSV (D), XBB.1.5 S VSV (E) and KP.2 S VSV (F) in the serum of individuals from the 2x XBB.1.5 S cohort (n= 15 samples), 2x XBB.1.5 S + 1x KP.2/JN.1 S cohort (n= 8 samples) or 1x XBB.1.5 S + 1x KP.2/JN.1 cohort (n= 11 samples) upon mock-depletion or depletion of Wu S-reactive antibodies. LOD: 20 (G-I) Antibody binding titers in the serum of patient ID 404, expressed as ED_50_, against Wu prefusion S (G), and XBB.1.5 or KP.2 prefusion S, RBD, NTD and prefusion S_2_ (H) and (I) SARS-CoV-2 variants. All individuals had previously received an XBB.1.5 S mRNA booster 80–396 days (median: 251 days) before the last booster. LOD: 80 for Wu, XBB.1.5 and KP.2 prefusion S and LOD: 20 for individual domains. (J) Prefusion SARS-CoV-2 Wu S structure (PDB: 6VXX^25^) low-pass filtered at 8 Å resolution highlighting the NTD (light blue), RBD (pink) and the S_2_ subunit trimer (blue, pink and yellow). In (A-C), each data point represents the ED_50_ for an individual obtained from the average of two biological replicates (using distinct protein batches), each comprising averaged technical duplicates. In (G-I), each data point represents the ED_50_ for individual 404C obtained from a biological replicate (each one using distinct protein batches), each comprising averaged technical duplicates. In (D-F), each data point represents the ID_50_ for an individual obtained from the average of two biological replicates (using distinct pseudovirus batches), each comprising averaged technical duplicates. Geometric mean titers (GMTs) are indicated. See also Figures S1, S2 and S3.

The greater (or comparable) neutralization potency observed for Wu/G614 VSV relative to other pseudoviruses evaluated in all cohorts and at all time points is a serological indication of immune imprinting^22^ given that all subjects received multiple doses of updated COVID-19 boosters (lacking Wu S). We thus set out to understand the contribution of recalled Wu S-cross-reactive antibodies to the serum binding and neutralizing activity against the XBB.1.5 and KP.2 variants following vaccination with XBB.1.5 and KP.2/JN.1 booster. We depleted polyclonal serum antibodies recognizing prefusion-stabilized Wu S and assessed remaining antibody binding titers against Wu S, XBB.1.5 S and KP.2 S ectodomain trimers using ELISAs. As expected, depletion of Wu S-directed antibodies markedly reduced Wu serum binding titers (Figures 2A and S2). We found that 5/9 (day 35) and 2/6 (day 97) individuals from the 2x XBB.1.5 cohort retain low but detectable levels of XBB.1.5 S-binding antibodies upon depletion (Figure 2B and S2). Furthermore, we observed that 4/4 (day 32) and 2/4 (day 111) individuals from the 2x XBB.1.5 S + 1x KP.2/JN.1 S cohort, and that 4/6 (day 22) and 3/5 (day 95) individuals from the 1x XBB.1.5 S + 1x KP.2/JN.1 S cohort retain detectable - albeit markedly dampened - XBB.1.5 S-binding antibodies upon depletion (Figures 2B and S2). Furthermore, 6/9 (day 35) and 3/6 (day 97) individuals from the 2x XBB.1.5 S cohort, 2/4 (day 32) and 3/4 (day 111) individuals from the 2x XBB.1.5 + 1x KP.2/JN.1 cohort, and 4/6 (day 22) and 4/5 (day 95) from the 1x XBB.1.5 + 1x KP.2/JN.1 cohort retain detectable levels of KP.2-directed antibodies after depletion (Figures 2C and S2). Out of all samples analyzed, only one patient (404C, 1x XBB.1.5 S + 1x KP.2/JN.1 S cohort) retained robust neutralizing activity after depletion of Wu S-directed serum antibodies. Indeed, depleted sera from patient 404C neutralized KP.2 S VSV, but not XBB.1.5 S VSV, at 22 and 95 days post vaccination (Figures 2D-F and S1), concurring with retention of high titers of KP.2 S-directed binding antibodies (Figures 2C and S2). To understand the specificity of antibodies targeting the KP2 and XBB.1.5 S trimers post depletion, we assessed binding to the KP.2- and XBB.1.5- RBDs, NTDs and prefusion-stabilized S_2_ subunit trimers^23^ using ELISA with selected sera (Figure 2G-J, S2 and S3). We found that 404C retained much more potent NTD-directed, and particularly RBD-directed, antibody responses against KP.2 at both time points, relative to XBB.1.5, concurring with prior observations that these two domains are the main targets of neutralizing antibodies^13–16,24^.

These findings show that administration of multiple updated COVID-19 boosters elicited variable magnitudes of polyclonal serum antibodies recognizing the XBB.1.5 and KP.2 S trimers without cross-reacting with Wu S. We suggest that immune imprinting is partially overcome through *de novo* elicitation of vaccine-matched antibody responses or redirection of previously existing antibody clones through affinity-maturation. However, serum neutralizing activity elicited by repeated booster administrations remains solely accounted for by a recall of Wu S cross-reactive antibodies induced by earlier exposures except for one individual (ID 404).

### Repeated booster administration increases the frequency of vaccine-matched memory B cells that do not cross-react with the Wu RBD

**Figure 3.**
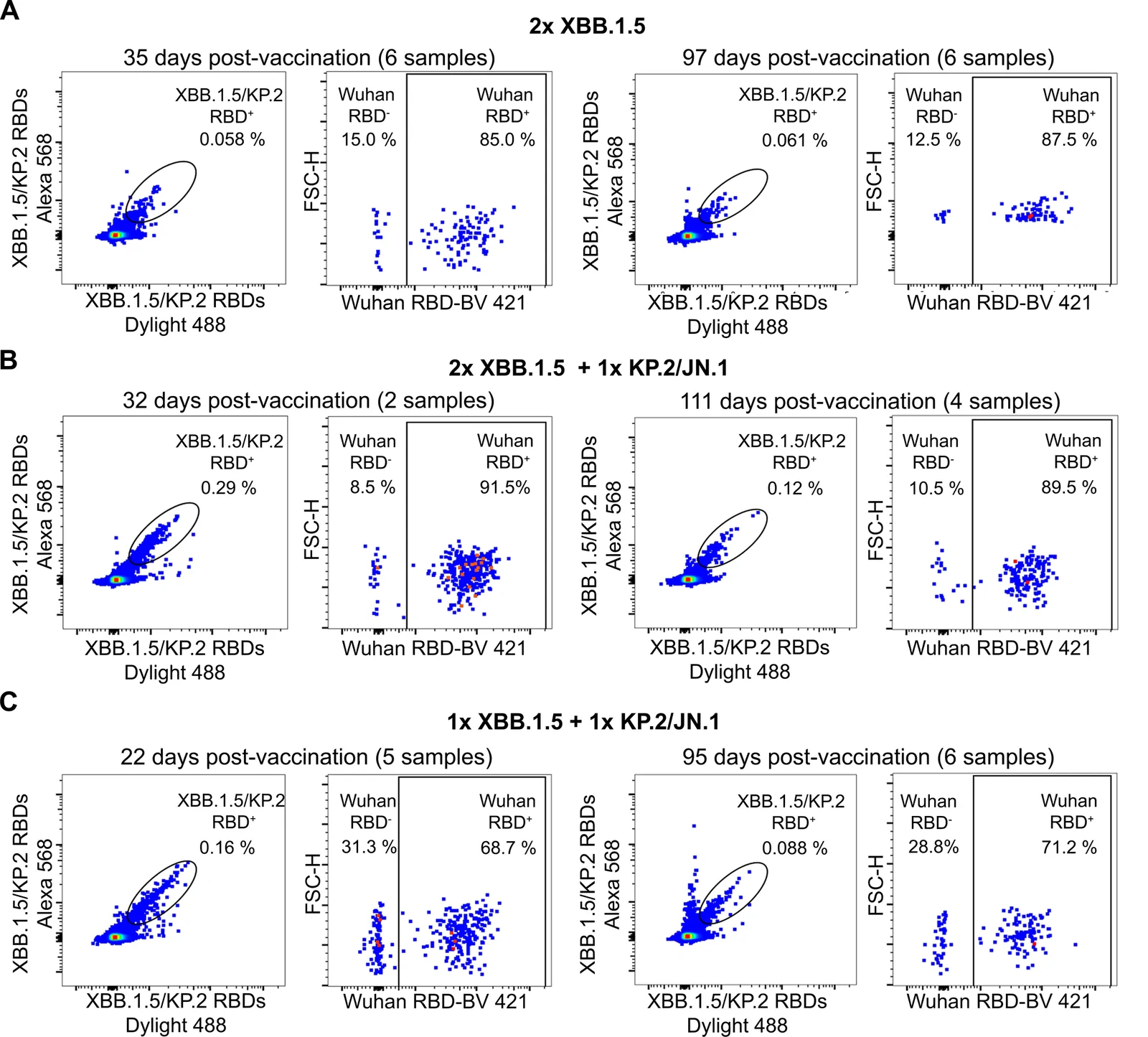
Booster administration increases the frequency of vaccine-matched memory B cells. (A-C). XBB.1.5/KP.2 RBD double-positive memory B cells (left) were analyzed for cross-reactivity with the Wuhan-Hu-1 RBD (right) using flow cytometry. Memory B cells were obtained from peripheral blood collected from individuals at 32–60 days (median: 35) or 87–163 days (median: 97) from the 2x XBB.1.5 cohort (A) at 31–33 days (median: 32) and 95–134 days (median: 111) from the 2x XBB.1.5 + 1x KP.2/JN.1 cohort (B) or at 12–33 days (median: 22) or 79–123 days (median: 95) from the 1x XBB.1.5 cohort + 1x KP.2/JN.1 cohort (C) See also Figure S4.

Prior work described large differences between the antigen-specific antibody repertoire in the memory compartment and serum antibody clonotype diversity and abundance^26–28^. To understand possible differences between these two compartments in the cohorts studied here, we analyzed memory B cell (MBC) populations in the peripheral blood at the same time points as those used for analysis of serum neutralizing activity. We used flow cytometry to enumerate the frequency of MBCs that reacted with an XBB.1.5/KP.2 RBD pool (XBB.1.5/KP.2 RBD^+^) and with the Wu RBD (Wu RBD^+^, Figure 3). XBB.1.5/KP.2 RBD^+^/Wu RBD^−^ MBCs accounted for a total of 15 % (day 35) and 12.5 % (day 97) for the 2x XBB.1.5 cohort, 8.5 % (day 32) and 10.5 % (day 111) for the 2x XBB.1.5 + 1x KP.2/JN.1 cohort, and 31.3 % (day 22) and 28.8 % (day 95) for individuals from the 1x XBB.1.5 + 1x KP.2/JN.1 cohort (Figure 3A-C). XBB.1.5/KP.2 RBD^+^/Wu RBD^−^ MBCs ranged in frequencies from 0 to 33.3 % (day 35) and 0 to 44.4 % (day 97) for the 2x XBB.1.5 cohort, 0–11.5 % (day 32) and 2.7–25.5 % (day 111) for the 2x XBB.1.5 + 1x KP.2/JN.1 cohort and from 0–52.7 % (day 22) and 15.8–33.3 % (day 95) for the 1x XBB.1.5 + 1x KP.2/JN.1 cohort (Figure S4). The total number of known S exposures (vaccination and infection) ranges between 5–9 for the 1x XBB.1.5 + 1x KP.2/JN.1 cohort and 11 for all but one individual (for whom it is 7) for the 2x XBB.1.5 + 1x KP.2/JN.1 cohort (Table 1). We observed XBB.1.5/KP.2 RBD^+^/Wu RBD^−^ MBCs in the majority of individuals across the current cohorts: 6 of 8 in the 2x XBB.1.5 group, 4 of 4 in the 2x XBB.1.5 + 1x KP.2/JN.1 group, and 5 of 6 in the 1x XBB.1.5 + 1x KP.2/JN.1 group (Figure S4). This contrasts with a previously described cohort receiving a single XBB.1.5 booster, where only 3 of 9 subjects displayed this phenotype^22^.

Our results point to a diversification of MBCs (to different extent for distinct individuals) in these cohorts through *de novo* elicitation of vaccine-matched memory B cells or redirection of previously existing clones through affinity-maturation.

## Discussion

The ability of the human immune system to recall memory B cells elicited by prior exposure protects individuals upon subsequent encounters with the same (or closely related) pathogens. Multiple safe and effective vaccines leveraging this property were developed to provide durable protection against several human pathogens, tremendously increasing life expectancy.

Immune imprinting, also known as original antigenic sin, was first observed in studies of human immunity to influenza virus and describes how the first exposure to a virus shapes immunological outcomes of subsequent exposures to antigenically related strains^18^. Although it is often considered detrimental, imprinting during childhood resulting from exposure to seasonal influenza H1N1 or H3N2 was proposed to respectively protect against H5N1 or H7N9 zoonotic strains, most likely via hemagglutinin stem-directed antibodies^29^. This was also the case during the 2009 H1N1 “swine flu” pandemic where initial antibody responses to infection in humans were dominated by antibodies targeting the conserved hemagglutinin stem region^30,31^.

Repeated exposures through infection and vaccination during the first few years of the COVID-19 pandemic resulted in widespread Wu S imprinting. Subsequent infection with immune-evasive SARS-CoV-2 Omicron variants along with vaccine updates to match the S glycoprotein sequence of antigenically-evolved variants did not overcome immune imprinting substantially^22,28,32–41^. Instead, humoral immunity remained dominated by recall of memory B cells and plasma antibodies targeting conserved antigenic sites among SARS-CoV-2 variants, which were elicited by prior exposure.

A study of subjects vaccinated twice with inactivated SARS-CoV-2 Wu vaccines followed by repeated Omicron infections reported substantial *de novo* antibody responses elicited against these newly emerged variants^42,43^. However, these findings appear to be specific to the cohort studied, possibly due to distinct vaccine platforms and total number of exposures. Although the exact mechanisms governing Wu S imprinting remain to be elucidated, prior studies have suggested that epitope masking by pre-existing antibodies can modulate subsequent antigen-specific antibody responses^44–46^.

Here, we report that repeated administration of updated vaccine boosters to individuals with Wu S imprinting and complex exposure history (representative of the global human population) elicits broadly neutralizing serum antibody responses against matched and mismatched SARS-CoV-2 variants. Furthermore, an appreciable fraction of memory B cells recognizing the updated vaccine RBDs, did not cross-react with the Wu RBD, indicating *de novo* priming of Omicron-specific naive B cells or redirection of pre-existing memory B cells leading to loss of Wu RBD binding^35,47^. We detected a higher fraction of XBB.1.5/KP.2 RBD^+^ MBCs that do not cross-react with the Wu RBD, relative to studies carried out after prior vaccine updates^22,32,35,47^, suggesting that immune imprinting is progressively overridden via MBC diversification. However, our depletion studies show that serum neutralizing activity is mediated by Wu S cross-reactive antibodies in all but one individual and that high titers of variant-specific, RBD-directed antibodies correlate with KP.2 neutralization in this latter case. These findings are consistent with a large body of data showing that RBD-directed antibodies account for most of the serum neutralizing activity against matched and mismatched SARS-CoV-2 variants^13–16 48–54^.

The rare detection of XBB.1.5 S- or KP.2 S-directed serum antibodies that do not cross-react with Wu S contrasts with the substantially higher fraction of memory B cells binding to the XBB.1.5/KP.2 RBD pool but not the Wu RBD in most individuals. These results point to differences between antibody composition and prevalence in the serum and memory compartments, suggesting that only few B cell clones give rise to long-lived bone marrow plasma cells responsible for producing serum antibodies^26,27,55^. Collectively, these findings suggest that continued administration of updated vaccine boosters may overcome immune imprinting, concurring with the elicitation of potent *de novo* plasma neutralizing antibodies after multiple doses of updated multivalent RBD-nanoparticle vaccine doses following mRNA-1273 Wu-imprinting in non-human primates^56^. Irrespective of modulation of immune imprinting, we note that clinical evaluation of updated COVID-19 booster vaccines established their continued effectiveness at reducing hospitalization and severe outcomes across populations, including older adults and the immunocompromised^57–61^, motivating continued reformulation and innovation to protect the human population widely.

## Limitations of the study

Our study is based on a relatively small sample size and only a fraction of the samples could be collected longitudinally from the same individuals, and the other samples were therefore obtained from different subjects at the different time points. Our analysis focused on class-switched (IgM^-^/IgD^-^) memory B cells which might have led to an underestimation of the fraction of B cells recognizing the XBB.1.5/KP.2 RBD pool without binding to the Wu RBD, as previously proposed^35^. Finally, serum antibodies and MBCs were analyzed for their reactivity to S and RBD antigens, respectively.

## Resource availability

### Lead Contact

Requests for further information and resources should be directed to and will be fulfilled by the lead contact, David Veesler (dveesler@uw.edu).

### Materials Availability

All unique/stable reagents generated in this study will be available on request from the lead contact with a complemented materials transfer agreement.

### Data and Code Availability

- All the data are presented in this manuscript.
- This manuscript does not report original code.
- Any additional information required to reanalyze the data reported here, is available from the lead contact upon request.

## STAR Methods

### Experimental model and study participant details

#### Cell culture and cell lines

VeroE6-TMPRSS2 (puromycin resistant) is a female cell line. Expi293F is a female cell line (Thermo Fisher). HEK293T is a female cell line (obtained from ATCC) and Il-mouse hybridoma is a female cell line (ATCC). Cells were cultivated at 37°C in an atmosphere of 5 % CO_2_ for adherent cells and 8 % CO_2_ with 130 rpm of agitation for suspension cells.

#### Cell line authentication

Cell lines used in this study were obtained from ATCC or Thermo Fisher and were not routinely tested for mycoplasma contamination.

#### Sample donors and collection

Sera and PBMCs were collected after informed consent from participants in the prospective longitudinal Hospitalized or Ambulatory Adults with Respiratory Viral Infections (HAARVI) study from Washington State, USA, which was approved by University of Washington Institutional Review Board (protocol #STUDY00000959). Demographic information is provided in Table 1.

#### Influence of sex and gender

Sex or gender variables were not a primary focus of this study. Where human or murine cell lines were used, sex of origin is known but was not considered as a variable.

### Method details

#### Plasmids

All genes used in this study were synthesized by GenScript, inserted in frame with a Kozak sequence to direct translation and codon optimized for expression in mammalian cells. Plasmid encoding for Wu S Hexapro ectodomain (residues 1–1208) and XBB.1.5 S Hexapro (residues 1–1203) were previously described^22^. KP.2 S Hexapro ectodomain (residues 1–1204) was cloned into pcDNA 3.1 (+) and, like constructs Wu S Hexapro and XBB.1.5 S Hexapro, harbors the HexaPro mutations (Hsieh et al., 2020), a wild-type signal peptide, a furin cleavage site mutated _685_RSV_687_ to _685_SSV_687_, an avi-tag and an octa-his tag for affinity purification.

The NTDs for XBB.1.5 and KP.2 (residues 14–304 for both strains, Wu numbering) and the RBDs for XBB.1.5 and KP.2 (residues 328–531 for both strains, Wu numbering) were cloned into pcDNA 3.1 (+) using the sequence MGILPSPGMPALLSLVSLLSVLLMGCVAETGT as signal peptide for all constructs except for the XBB.1.5 RBD in which the sequence MARAWIFFLLCLAGRALA was used as signal peptide. At the C-terminal region of the domains were inserted an avi-tag and an octa-his tag for affinity purification. Constructs for membrane-anchored S glycoproteins for SARS-CoV-2 Wu/G614 and XBB.1.5 were previously described^22^.

Plasmid encoding for the SARS-CoV-2 S_2_ subunit harboring prefusion-stabilizing mutations was previously described ^23^. The S_2_ prefusion-stabilizing mutations designed for SARS-CoV-2 Wu S_2_ (Y707C, T883C, A892P, A899P, N907E, A942P, T961F, F970C, V987P, D994Q, G999C, Q10 11M and I1018Y) were incorporated in synthetic genes encoding the XBB.1.5 and KP.2 S_2_ (residues 701–1208 for both constructs, Wu numbering) cloned into pcDNA 3.1 (+) using the sequence MGILPSPGMPALLSLVSLLSVLLMGCVAETGT as signal peptide and at the C-terminal a GGS linker followed by a TEV cleavage site, a GGGS linker, an avi-tag and an deca-his tag for affinity purification.

Membrane-anchored S glycoprotein constructs for JN.1 (residues 1–1248), KP.2 (residues 1–1248) and KP.3 (residues 1–1248) were all cloned into an HDM vector with the authentic signal peptide and harboring a C-terminal deletion of 21 residues^63^. Mutations included in the variants XBB.1.5, JN.1, KP.2 and KP.3 are described in Table 2.

#### Recombinant protein production

To express Wu, XBB.1.5, KP.2 stabilized S ectodomains with the hexapro mutations, NTDs, RBDs and stabilized S_2_ ectodomains, Expi293 cells were transfected with the corresponding plasmids using Expifectamine following the manufacturer’s instructions. Four-five days post-transfection, supernatants were clarified by centrifugation at 4,121 × g for 30 minutes, supplemented with 25 mM sodium phosphate pH 8.0, 300 mM NaCl. Supernatant was vacuum filtered (0.22 µm) and mixed with Ni Sepharose Excel resin (Cytiva), previously equilibrated in 25 mM sodium phosphate pH 8.0, 300 mM NaCl. After 2 h incubation at room temperature in a roller shaker, supernatant was added to a gravity flow column and washed with 25 mM sodium phosphate pH 8.0, 300 mM NaCl and 20–40 mM imidazole. Proteins were eluted with 25 mM sodium phosphate pH 8.0, 300 mM NaCl and 300–500 mM imidazole and buffer exchanged in 50 mM Tris-HCl pH 7.4, 150 mM NaCl using a centrifugal device (Amicon) with the proper MWCO according to the molecular weight and stored at −80°C or immediately used. Wu, XBB.1.5 and KP.2 S_2_, NTDs and RBDs were further purified at room temperature by size-exclusion chromatography (SEC) using a Superose 6 Increase 10/300 GL for the S_2_ proteins and Superdex 200 Increase 10/300 GL for NTDs and RBDs, previously equilibrated in 50 mM Tris-HCl pH 7.4, 150 mM NaCl. Protein fractions were pooled and flash-frozen in liquid nitrogen and stored at −80°C until use.

#### Negative stain electron microscopy

XBB.1.5 and KP.2 stabilized prefusion S_2_ were adsorbed to glow-discharged carbon-coated copper grids for 30 s prior to 2 % uranyl formate staining. Micrographs were recorded using the Leginon software^64^ on a 120 kV FEI Tecnai G2 Spirit with a Gatan Ultrascan 4000 CCD camera at 67,000 nominal magnification. The defocus ranged from 1.0 to 2.0 µm, and the pixel size was 1.6 Å.

#### Sera antibody depletion

Invitrogen His-Tag Dynabeads (Thermo Fisher) were used for depletion of serum samples from antibodies recognizing the Wu S trimer, as previously described^22^ with some modifications. Vortexed beads were incubated at room temperature on a DynaMag-2 Magnet (Thermo Fisher) for 2 min to allow beads to separate for the liquid phase. Supernatant was discarded and beads were washed one time with TBS-T (20 mM Tris-HCl pH7.5, 150 mM NaCl, 0.1 % (w/v) Tween 20) and divided in two tubes. After a 2 min incubation on the magnet, TBS-T supernatants were discarded, and one set of beads was incubated with 4 mg of purified his-tagged Wu prefusion S stabilized ectodomain trimer in purification buffer (Wu S depletion) and the other set was incubated with TBS-T alone (mock depletion) with gentle rotation for 1 h at room temperature. Supernatants were discarded using the magnet and beads were washed three times with TBS-T. Subsequently, 20 ml of each of the sera samples were incubated with 80 ml of S-loaded beads or mock-loaded beads for 1 h at 37°C during which time they were mixed every 15 min. Sera samples were separated from the beads using the magnet and subjected to a second round of depletion using the same protocol as described for the first round. Incubated supernatants were recovered using a magnet to separate them from the beads and used for neutralization and binding assays.

#### Enzyme-linked immunosorbent assays (ELISA)

Analysis of sera binding antibodies for samples mock-depleted or depleted of antibodies binding to the Wu S ectodomain trimer was performed using ELISAs. Briefly, clear flat bottom Immuno Nonsterile 384-well plates (Thermo Fisher) were coated overnight at room temperature with 30 µl of the indicated antigens prepared at 3 ng/ml per well in PBS (137 mM of NaCl, 2.7 mM of KCl, 10 mM of Na_2_HPO4, and 1.8 mM of KH_2_PO_4_, pH 7.2). The next day, plates were blocked with Blocker Casein (Thermo Fisher) at 37 C and subsequently incubated with serial dilutions of sera samples for 1 h at 37°C. After four washing steps with TBS-T, goat anti-human IgG-Fc secondary antibody conjugated to HRP (Thermo Fisher), diluted 1/500 was added and incubated for 1 h at 37°C. Plates were washed four times with TBS-T and KPL SureBlue Reserve TMB Microwell Peroxidase Substrate (VWR) was added. After 2 min incubation, 1N HCl was added and absorbance at 405 nm was measured using a Biotek Neo2 plate reader. Data were plotted using Prism 10 (GraphPad).

#### Protein biotinylation

To generate RBDs-streptavidin tetramers for flow cytometry analysis, the RBDs were biotinylated using BirA biotin-protein ligase standard reaction kit (Avidity) following manufacturer’s protocol. In a typical reaction, 1 mg of RBD is incubated with 1–2 µg of BirA enzyme overnight at 4°C in reaction mixtures containing 1X BiomixB, 1X BiomixA and 40 mM BIO200. Proteins were further separated from Bir A by SEC using Superdex 200 increase 10/300 GL or Superose 6 increase 10/300 GL (GE LifeSciences) for RBDs or spikes, respectively, previously equilibrated in 50 mM Tris-HCl pH 8.0, 150 mM NaCl and concentrated using either 30 kDa for RBDs or 100 kDa filters (Amicon) for spikes. Proteins fractions were pooled and flash-frozen in liquid nitrogen and stored at −80°C until use.

#### Flow cytometry analysis of SARS-CoV-2 RBD-reactive memory B cells

To define specific B cell populations reactive with the XBB.1.5, KP.2 and Wu RBDs, RBD-streptavidin tetramers conjugated to fluorophores were generated by incubating the corresponding biotinylated RBDs with streptavidin at a 4:1 molar ratio for 30 min at 4°C. Excess of free biotin was added to the reaction to bind any unconjugated sites in the streptavidin tetramers. The RBD-streptavidin tetramers were washed once with PBS (137 mM NaCl, 2.7 mM KCl, 10 mM Na_2_HPO4, 1.8 mM KH_2_PO4, pH 7.4) and concentrated with a 100-kDa cut-off centrifugal concentrator (Amicon). An additional streptavidin tetramer conjugated to only biotin was generated and included in the staining as decoy. Approximately 5 to 10 million PMBCs were collected at the indicated times for individuals who received vaccine boosters. Cells were collected by centrifugation at 1000 g for 5 min at 4°C, washed twice with PBS and stained with Zombie Aqua dye (Biolegend) diluted 1:100 in PBS at room temperature. After 30 min incubation, cells were washed twice with PBS and stained with antibodies for CD20-PECy7 (BD), CD3-Alexa eFluor780 (Thermo Fisher), CD8-Alexa eFluor780 (Thermo Fisher), CD14-Alexa eFluor780 (Thermo Fisher), CD16-Alexa eFluor780 (Thermo Fisher), IgM-Alexa Fluor 647 (BioLegend), IgD-Alexa Fluor 647 (BioLegend), and CD38-Brilliant Violet 785 (BioLegend), all diluted 1:200 in Brilliant Stain Buffer BD), along with the RBD- or spike-streptavidin tetramers for 30 min at 4°C. Cells were washed three times, resuspended in PBS, and passed through a 5 ml Polystyrene round-bottom tube with cell-strainer cap (Falcon) before being examined on a BD FACSymphony A3 for acquisition and FlowJo 10.8.1 for analysis. Gates for identifying the XBB.1.5 or KP.2 RBD double-positive population as well as the subsequent Wu RBD-positive and Wu RBD-negative populations were drawn based on staining of fluorescent minus one as controls.

#### VSV pseudotyped virus production and neutralization

Vesicular stomatitis virus (VSV) was pseudotyped with SARS-CoV-2 Wu S harboring the G614, XBB.1.5, KP.2, KP.3 or JN.1 mutations following a previously described protocol (Tortorici et al., 2024). Briefly, HEK293T cells seeded in poly-D-lysine-coated 10-cm dishes in DMEM (Gibco, Thermo Fisher) supplemented with 10 % FBS and 1 % PenStrep were transfected with 24 µg of the corresponding plasmid, 60 µl Lipofectamine 2000 (Life Technologies) in 3 ml of Opti-MEM (Thermo Fisher), following the manufacturer’s instructions. Transfection mixture was added to 10 cm plates containing washed cells and 7mL of DMEM and 10 % FBS. The next day, cells were washed three times with DMEM and were transduced with VSV ∆G-luc.79 After 2 h, the virus inoculum was removed and cells were washed five times with DMEM prior to the addition of DMEM supplemented with anti-VSV-G antibody [Il-mouse hybridoma supernatant diluted 1 to 25 (v/v), from CRL-2700, ATCC] to minimize parental background. After 18–24 h, supernatants containing pseudotyped VSV were harvested, centrifuged at 2,000 × g for 10 min to remove cellular debris, filtered with a 0.45 mm membrane, concentrated 10 times using a 30 kDa cut-off membrane (Amicon), aliquoted, and frozen at −80°C.

For VSV pseudotyped neutralizations, VeroE6-TMPRSS2 cells were seeded into coated clear bottom white walled 96-well plates at 20,000 cells/well and cultured overnight at 37°C. Twelve 3-fold serial dilutions of each sera sample were prepared in DMEM. Pseudotyped VSV viruses, diluted 1 to 10 in DMEM containing anti-VSV-G antibody [Il-mouse hybridoma supernatant diluted 1 to 25 (v/v), from CRL-2700, ATCC], were added 1:1 (v/v) to each sera sample dilution and mixtures of 40 µl volume were incubated for 45–60 min at room temperature. VeroE6-TMPRSS2 cells were washed three times with DMEM and the mixtures pseudovirus/ sera dilutions were added to the cells. Two hours later, 40 µl of DMEM were added to the cells. After 17–20 h, 40 µl of One-Glo-EX substrate (Promega) were added to each well and incubated on a plate shaker in the dark at 37°C. After 5–15 min incubation, plates were read on a Biotek Neo2 plate reader. Measurements were done in duplicate with at least two biological replicates. Relative luciferase units were plotted and normalized in Prism 10 (GraphPad): cells without pseudotyped virus added were defined as 0 % infection or 100 % neutralization, and cells with virus only (no sera) were defined as 100 % infection or 0 % neutralization.

### Quantification and statistical analysis

Statistical analysis was performed only for Figure 1 in which the comparison of individual ID_50_ values for each pseudovirus (JN.1, KP.2 or KP.3) was done using the 2x XBB.1.5 S cohort as reference for the first (black labels) and second (blue labels) blood draws independently with one-way analysis of variance (ANOVA). Not significant (ns)=P≥0.05, *P=0.01 to 0.05 and **P=0.001 to 0.01 and the results are presented as mean ± SD. Information about the quantification for individual experiments are included in the figure legends.

### Additional resources

This study did not generate any new unique reagents.

## Supplementary Material

Supp Figures

Supplemental Information

Document S1. Supplemental Figures 1-4.

## Figures and Tables

**Table 1.** Demographics information of the cohort participants. Total number of exposures correspond to infection and vaccination.

ID	Sex	Age	Ethnicity	Identify as Hispanic or Latino?	# of pre-Omicron infections	# of post-Omicron infection	# of Wu doses	# of Bivalent (Wu/BA.5) doses	# of XBB.1.5 doses	# of KP2 doses	# of JN1 doses	# of KP2 or JN1 doses (unknown vaccine type)	Total number of exposures	Days between XBB.1.5 2^nd^ Vaccine and blood draw	Days between KP2, JN1 or KP2/JN1 Vaccine and blood draw
29C	F	46	White	No	1	2	4	1	2	0	1	0	11	35, 103	32
87C	M	81	White	No	1	1	4	2	2	1	0	0	11	32, 87	31, 108
106C	F	66	White	No	1	1	3	1	1	1	0	0	8		33, 93
108C	F	69	White	No	1	2	4	1	2	1	0	0	11	60, 163	113
172C	F	77	White	No	1	1	3	0	2	0	0	0	7	36, 97	
183C	M	71	White	No	1	0	3	0	2	1	0	0	7	41, 89	33,95
329C	M	65	Hebrew	No	1	1	3	0	2	0	0	0	6	36	
385C	F	33	White	No	1	1	3	1	1	1	0	0	8		16, 79
404C	M	58	White	No	1	1	4	1	1	1	0	0	9		27, 123
437C	M	77	White	No	0	1	4	2	2	0	0	0	9	34, 101	
459C	M	71	White	No	0	1	4	2	2	0	0	0	9	32	
485C	F	43	White	No	0	1	4	1	2	0	0	0	8	34, 94	
503C	M	53	White	No	0	2	4	2	2	0	1	0	11		31, 134
563C	F	56	White	No	0	1	4	1	1	1	0	0	8		30
530C	F	12	White	No	0	1	2	0	1	0	0	1	5		12, 95
531C	F	15	White	No	0	1	2	0	1	0	0	1	5		12, 95

**Table 2.** Mutations in the SARS-CoV-2 variants XBB.1.5, JN.1, KP.2 and KP.3 S full-length relative to Wuhan-Hu-1/G614 S full-length. Ectodomains of the same variant genes contain in addition the hexapro mutations ^62^. Residue numbering is based on Wuhan-Hu-1/G614 S.

Variant	Mutations
XBB.1.5	T19I, L24del, P25del, P26del, A27S, V83A, G142D, Y144del, H146Q, Q183E, V213E, G252V, G339H, R346T, L368I, S371F, S373P, S375F, T376A, D405N, R408S, K417N, N440K, V445P, G446S, N460K, S477N, T478K, E484A, F486P, F490S, Q498R, N501Y, Y505H, H655Y, N679K, P681H, N764K, D796Y, Q954H, N969K
JN.1	17M, L18P, T19L, F20ins, T20N, R21L, T22I, Q23T, L24T, P25T, P26Q ,A27S, S50L, H69del, V70del, V127F, G142D, Y144del, F157S, R158G, N211del, L212I, V213G, L216F, H245N, A264D, I332V, G339H, K356T, S371F, S373P, S375F, T376A, R403K, D405N, R408S, K417N, N440K, V445H, G446S, N450D, L452W, L455S, N460K, S477N, T478K, N481K, V483del, E484K, F486P, Q498R, N501Y, Y505H, E554K, A570V, P621S, H655Y, I670V, N679K, P681R, N764K, D796Y, S939F, Q954H, N969K, P1143L
KP.2	JN.1 mutations plus F456L, R346T, V1104L
KP.3	JN.1 mutations plus F456L, Q493E, V1104L

**Table:** Key Resources table

REAGENT or RESOURCE	SOURCE	IDENTIFIER
**Antibodies**		
CD20-PECy7	BD Biosciences	Cat# 335793; RRID:AB_399971
CD3-Alexa eFluor780	Thermo Fisher	Cat# 47-0037-41; RRID:AB_2573935
CD8-Alexa eFluor780	Thermo Fisher	Cat# 47-0086-42; RRID:AB_2573945
CD14-Alexa eFluor780	Thermo Fisher	Cat# 47-0149-42; RRID:AB_1834358
CD16-Alexa eFluor780	Thermo Fisher	Cat# 47-0168-41; RRID:AB_11219083
IgM-Alexa Fluor 647	BioLegend	Cat# 314535; RRID:AB_2566612
IgD-Alexa Fluor 647	BioLegend	Cat# 348227; RRID:AB_2563268
CD38_Brilliant Violet 785	BioLegend	Cat# 303529; RRID:AB_2561368
Brilliant Violet 421 Mouse IgG	BioLegend	Cat# 400157
BV11 Mouse anti-human IgG	BD OptiBuild	Cat# 740796
Alexa Fluor 568 Rabbit IgG monoclonal	Abcam	Cat# 1045530-17
Mouse IgG1 Dylight 488	Thermo Fisher	Cat# MA1-191-D488
Goat anti-Human IgG FC Cross-Adsorbed Secondary Antibody, HRP	Thermo Fisher	Cat# A18823
**Bacterial and virus strains**		
E.*coli* DH10B competent cells	Invitrogen	Cat# 44-0099
**Chemicals, peptides, and recombinant proteins**		
Blocker Casein	Thermo Fisher	Cat# J600289.AP
Lipofectamine 2000 Transfection Reagent	Invitrogen	Cat# 11668027
SureBlue Reserve TMB Microwell Peroxidase Substrate, KPL	VWR	Cat# 5120-0083
Flow Cytometry Staining Buffer	Thermo Fisher	Cat# 00-4222-26
ExpiFectamine 293 Transfection Kit	Thermo Fisher	Cat# A14525
Zombie Aqua dye	Biolegend	Cat# 423101
Brilliant Stain Buffer	BD Biosciences	Cat# 563794
PBS pH 7.4	Gibco	Cat# 10010-031
Penicillin-Streptomycin	Thermo Fisher	Cat# 15140163
Gibco Trypsin-EDTA 0.25 %	Thermo Fisher	Cat# 25200056
Fetal Bovine Serum	Thermo Fisher	Cat# S3039603HI
Gibco DMEM, high glucose	Thermo Fisher	Cat# 11-965-118
Opti-MEM	Thermo Fisher	Cat# 31985088
Brilliant Violet 711 Streptavidin	BioLegend	Cat# 405241
Brilliant Violet 421 Streptavidin	BioLegend	Cat# 405226
DyLight 488 Streptavidin	BioLegend	Cat# 405218
Streptavidin, Alexa Fluor 568	Thermo Fisher	Cat# S11226
**Critical commercial assays**		
One-Glo EX Luciferase Assay System	Promega	cat# E8150
BirA biotin-protein ligase standard reaction kit	Avidity	Cat# 341113
**Experimental models: Cell lines**		
HEK293T cells	ATCC	cat# CRL-11268
VeroE6-TMPRSS2 (puromycin resistant)	Lempp et al. 2021	https://www.nature.com/articles/s41586-021-03925-1
Expi293F cells	Thermo Fisher	cat# A14527
Il-mouse hybridoma	ATCC	CRL-2700
**Recombinant DNA**		
VSV (G*∆G-luciferase) DNA	Kaname et al. 2010	https://journals.asm.org/doi/10.1128/jvi.02519-09
pcDNA3.1(-): Wu S Hexapro ectodomain	Tortorici et al. 2024	https://www.sciencedirect.com/science/article/pii/S107476132400092X?via%3Dihub
pcDNA3.1(+): XBB.1.5 S Hexapro ectodomain	Tortorici et al. 2024	https://www.sciencedirect.com/science/article/pii/S107476132400092X?via%3Dihub
HDM vector: Wu/G614 S Full-length	Bowen et al. 2022	https://www.science.org/doi/10.1126/science.abq0203
HDM vector: XBB.1.5 S Full-length	Tortorici et al. 2024	https://www.sciencedirect.com/science/article/pii/S107476132400092X?via%3Dihub
HDM vector: JN.1 S Full-length	Tortorici et al. 2024	https://www.sciencedirect.com/science/article/pii/S107476132400092X?via%3Dihub
pcDNA3.1(+): KP.2 S Full-length	GeneScript	This study
HDM vector: KP.3 S Full-length	GeneScript	This study
pcDNA3.1 (-): Wu S RBD	Walls et al. 2020	https://www.sciencedirect.com/science/article/pii/S0092867420302622?via%3Dihub
pcDNA3.1(+): XBB.1.5 S RBD	Addetia et al. 2023	https://www.nature.com/articles/s41586-023-06487-6
pCDNA3.1(+): KP.2 S RBD	GeneScript	This study.
pCDNA3.1(+): XBB.1.5 S_2_	GeneScript	This study.
pCDNA3.1(+): KP.2 S_2_	GeneScript	This study.
pCDNA3.1(+): XBB.1.5 NTD	GeneScript	This study.
pCDNA3.1(+): KP.2 NTD	GeneScript	This study.
**Software and algorithms**		
FlowJo 10.8.1	BD Biosciences	https://www.flowjo.com/
BioRender	N/A	https://www.biorender.com/
Prism 10	GraphPad	https://www.graphpad.com/
**Other**		
Copper grids	Ted Pella	Cat# 01754-F
Uranyl formate	Electron Microscope Science	Cat#16984-59-1
Ultracomp eBeads plus	Thermo Fisher	Cat# 01-3333-42
ArC reactive beads	Thermo Fisher	Cat# A10346 A
ArC negative beads	Thermo Fisher	Cat# A10346 B
poly-D-lysine-coated 10 cm dishes	Thermo Fisher	Cat# 08-757-466
Immuno Nonsterile 384-well plates	Thermo Fisher	Cat# 464718
Costar Assay Plate, 96 well white with clear flat bottom	Corning	Cat# 3903
EndoFree Plasmid Mega kit	Qiagen	Cat# 12381
His-Tag Dynabeads	Thermo Fisher	Cat# 10104D
DynaMag-2 Magnet	Thermo Fisher	Cat# 12-321-D
100-kDa cut-off concentrator	Amicon	Cat# UFC910024
30-kDa cut-off concentrator	Amicon	Cat# UFC903096
10-kDa cut-off concentrator	Amicon	Cat# UFC901096
Sepharose excel histidine-tagged protein purification resin	Cytiva	Cat# 17371203
Superdex 200 Increase 10/300 GL	Cytiva	Cat# 28990944
Polystyrène round-bottom tube with cell-strainer cap	Falcon, a corning brand	Cat# 352235

## Highlights

- XBB.1.5, JN.1 and KP.2 S vaccine boosters elicit neutralizing antibodies against current variants.
- Neutralizing antibodies against current variants largely derive from recall of Wu S responses.
- Antibodies specific for updated S antigens were detected after multiple updated boosters.
- Variant-specific neutralizing antibodies predominantly target the RBD.
