# Supplementary material for: Repeated COVID-19 vaccine boosters elicit variant-specific memory B cells in humans
Source: Cell Rep. Author manuscript; Available in PMC 2026 Sep 24. (PMC13607844; doi:10.1016/j.celrep.2026.117052)
Supplement: Supplementary file 1 [file NIHMS2210869-supplement-Supp_Figures.pdf]

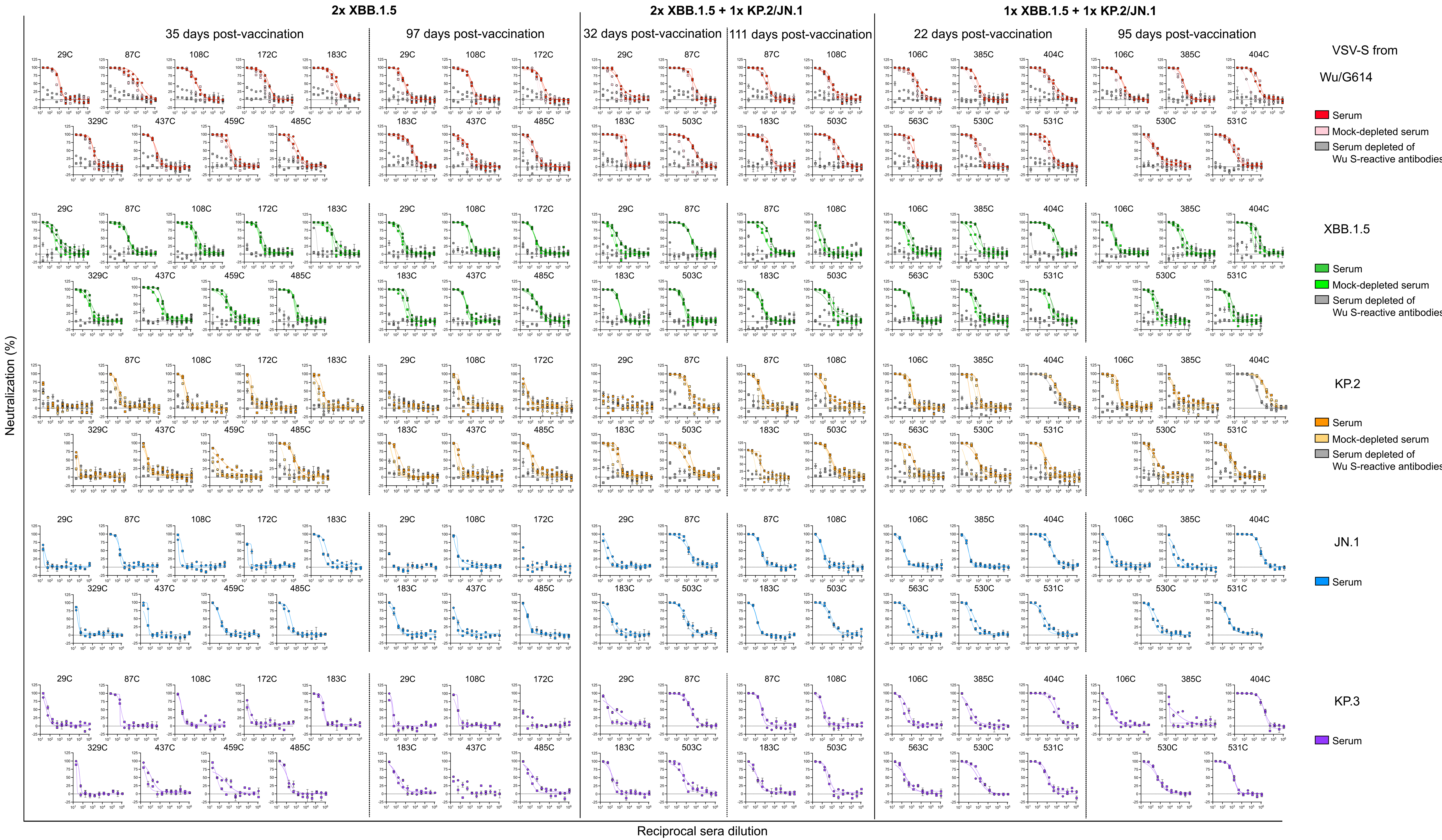

Supplementary Figure 1, related to Figure 1. Serum neutralizing antibody titers after vaccination with XBB.1.5, JN.1 or KP.2 COVID-19 boosters. Dose-response neutralization curves of Wu/G614 S, XBB.1.5 S, KP.2 S, JN.1 S and KP.3 S VSV pseudoviruses mediated by human sera (Figure 1) and by human sera that was either mock-depleted or depleted of Wu S-reactive antibodies (Figure 2). Each curve represents one biological replicate each performed with technical duplicates. Error bars represent the SEM from two technical replicates shown using squares symbols with dashed lines or with circles symbols and solid lines. Cohort member IDs are indicated on top of each graph. LOD: 20. If neutralizing activity reaches 50 % for only one of the two repeats, the ID_{50} value for this sample was set to the LOD.

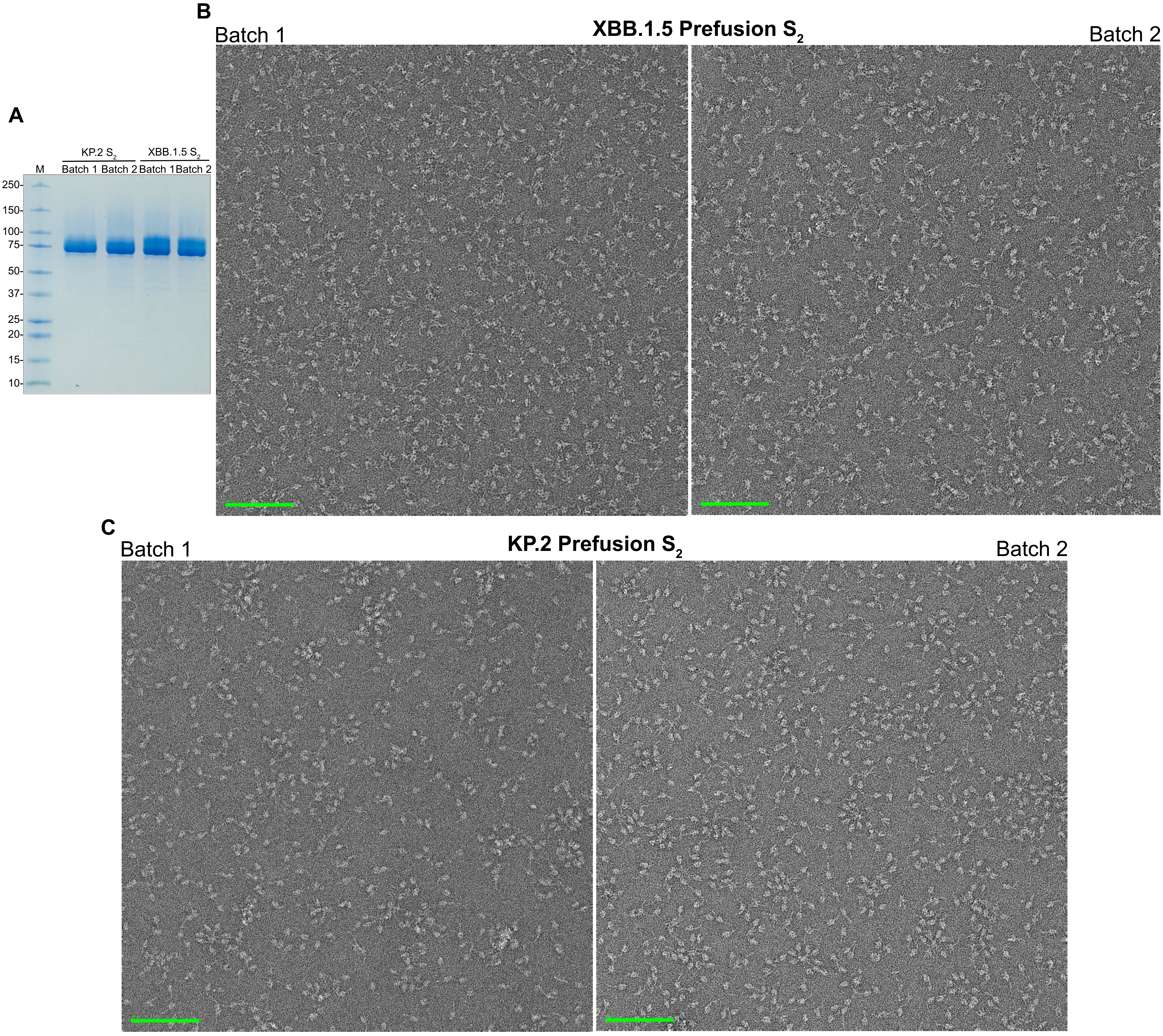

Supplemental Figure 2, related to Figure 2. Characterization of prefusion-stabilized XBB.1.5 and KP.2 S2 trimers. (A) SDS-PAGE. (B, C) Electron microscopy analysis of negatively stained purified XBB.1.5 (B) and KP.2 (C) prefusion-stabilized S_2 ectodomain trimers. Scale bars: 200 nm.

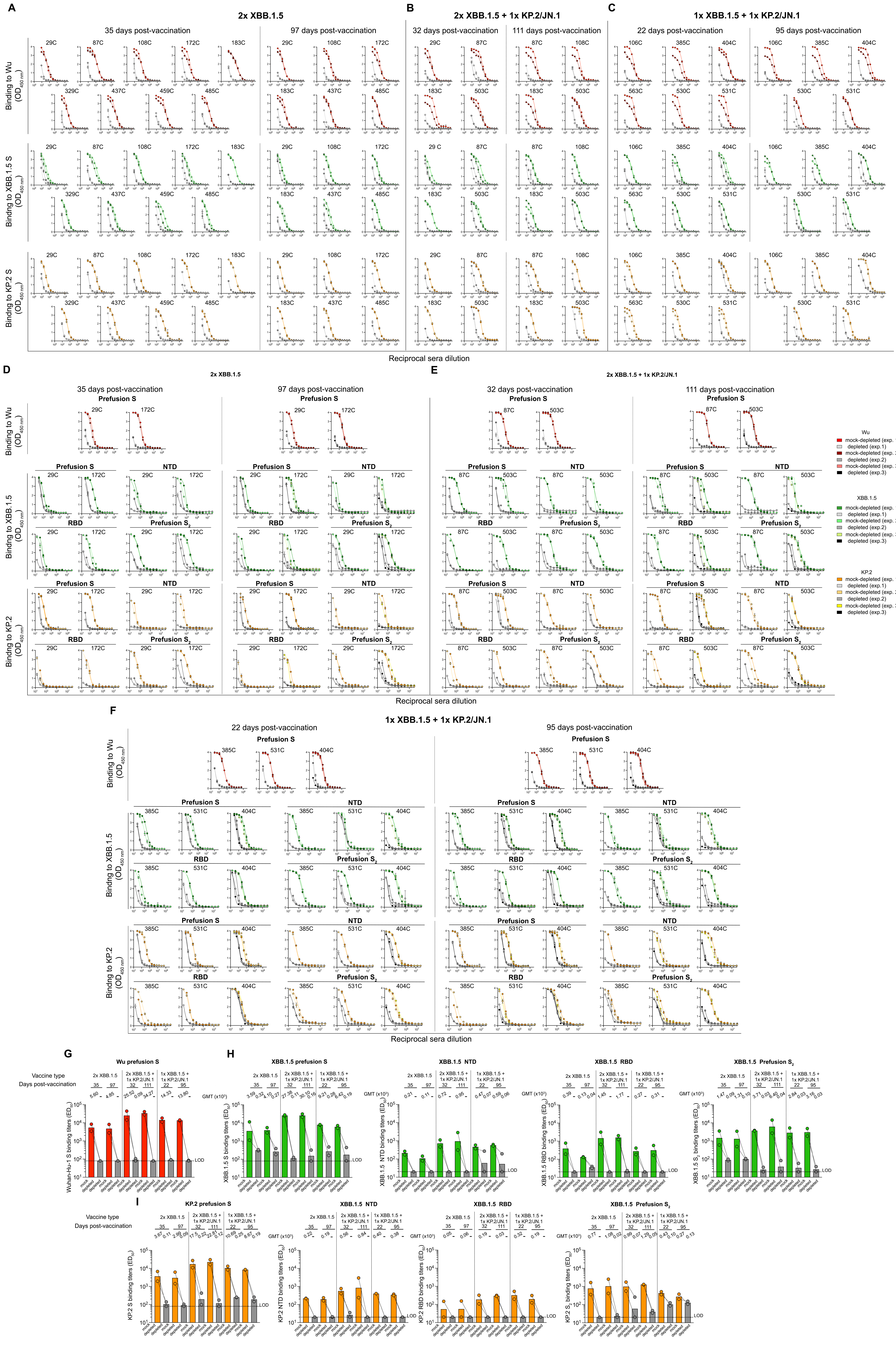

Supplementary Figure 3, related to Figure 2. Serum antibody binding titers after vaccination with XBB.1.5, JN.1 or KP.2 COVID-19 boosters. (A-C) ELISA dose-response curves for analysis of binding to prefusion Wu S, XBB.1.5 S and KP.2 S by human sera that was either mock-depleted or depleted of Wu S-reactive antibodies. (D-F) ELISA dose-response curves for analysis of binding to prefusion Wu S, XBB.1.5 S and KP.2 S, XBB.1.5 and KP.2 RBDs, NTDs and perfusion-stabilized S2 ectodomain trimers by human sera that was either mock-depleted or depleted of Wu S-reactive antibodies. Only sera retaining binding to XBB.1.5 S or KP.2 S post depletion were analyzed. Each curve represents one biological replicate each performed with technical duplicates. Error bars represent the SEM from two technical replicates Cohort member IDs are listed above each graph. (G-I) Antibody binding titers, expressed as ED_{50} values, against prefusion Wu S (G), XBB.1.5 S and KP.2 S, XBB.1.5 and KP.2 RBDs, NTDs and prefusion-stabilized S2 ectodomain trimers (H-I) for sera retaining binding to XBB.1.5 S or KP.2 S post depletion were analyzed. Each data point represents the ED_{50} for an individual obtained from one biological replicate (using distinct pseudovirus batches), each comprising an average of technical duplicates. GMTs are indicated. LOD: 80 for prefusion S and LOD: 20 for individual domains.

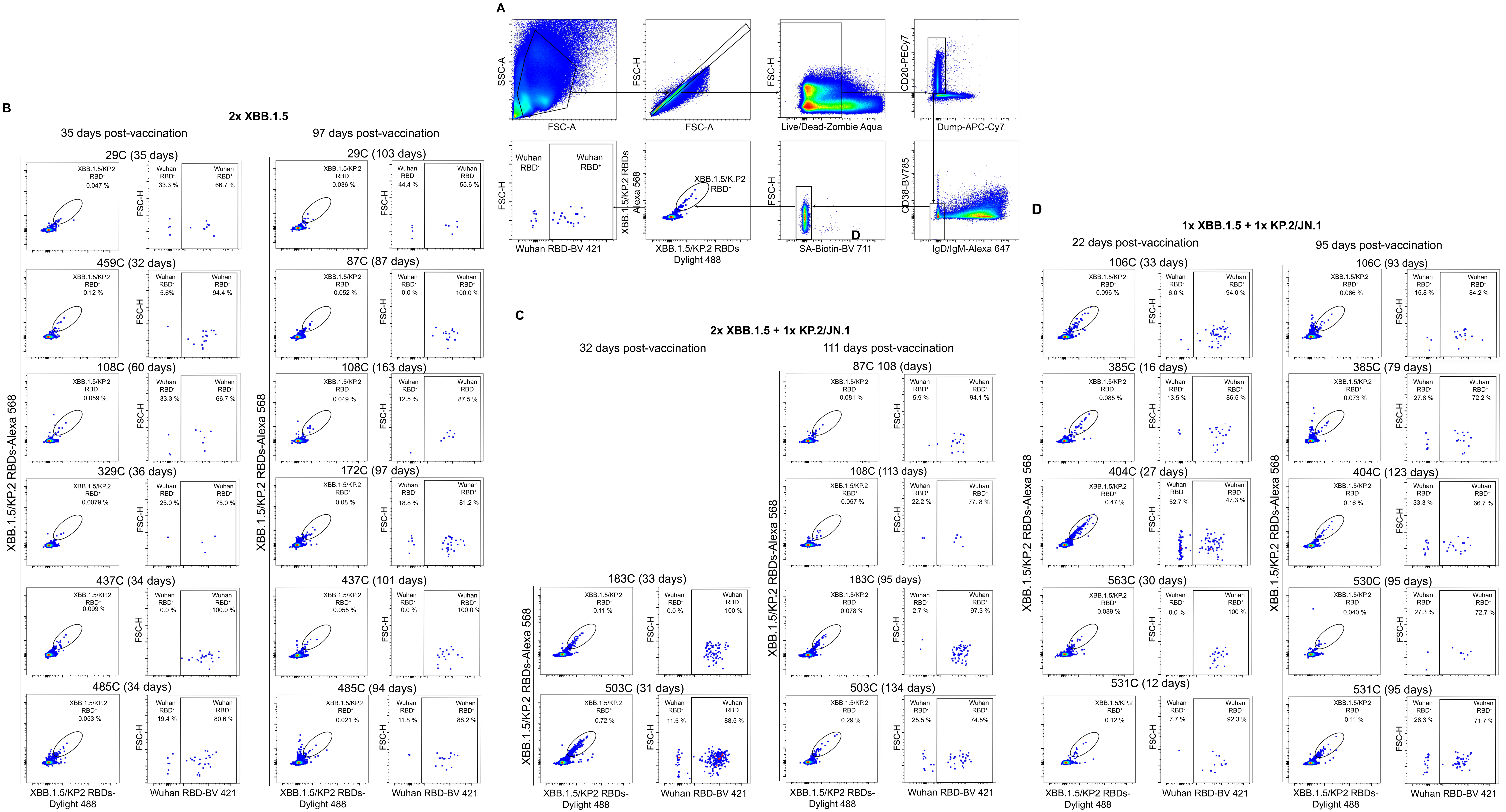

Supplemental Figure 4, related to Figure 3. Flow cytometry analysis of memory B cells. (A) Gating strategy to evaluate the cross-reactivity of Wuhan-Hu-1 RBD memory B cells binding to the XBB.1.5/KP.2 RBD pool. Dump includes markers for CD3, CD8, CD14, and CD16. The sample shown corresponds to ID: 404C, cohort 1x XBB.1.5 + 1x KP.2/JN.1, 95 days post-vaccination. (B). Gating of XBB.1.5/KP.2 S RBD-reactive memory B cells and subsequent evaluation of Wu S RBD-binding of these memory B cells from peripheral blood of each individual collected at the indicated times from the 2x XBB.1.5 S (B), the 2x XBB.1.5 + 1x KP.2/JN.1 S (C) or 1x XBB.1.5 + 1x KP.2/JN.1 (D) cohorts using flow cytometry. The analysis was performed once.
